# Real-World Efficacy of Transarterial Embolization and Transarterial Chemoembolization in Unresectable Hepatocellular Carcinoma: a Nationwide Cohort Study in Taiwan

**DOI:** 10.7150/ijms.119821

**Published:** 2026-01-01

**Authors:** Yu-Han Huang, Ping-Jen Hu, Sung-Hua Chiu, Wei-Chou Chang, Yuh-Feng Lin, Po-Ya Chang

**Affiliations:** 1 Department of Radiology, Tri-Service General Hospital, National Defense Medical University, Taipei, Taiwan.; 2 Department of Internal Medicine, Division of Gastroenterology, Shuang Ho Hospital, Taipei Medical University, New Taipei City, Taiwan.; 3 TMU Research Center for Digestive Medicine, Taipei Medical University, Taipei, Taiwan.; 4 Graduate Institute of Clinical Medicine, College of Medicine, Taipei Medical University, Taipei, Taiwan.; 5 Graduate Institute of Medical Sciences, National Defense Medical University, Taipei, Taiwan.; 6 Division of Nephrology, Department of Internal Medicine, Tri-Service General Hospital, National Defense Medical University, Taipei, Taiwan.; 7 Department of Leisure Industry and Health Promotion, National Taipei University of Nursing and Health Sciences, Taipei, Taiwan.

**Keywords:** hepatocellular carcinoma, transarterial embolization, transarterial chemoembolization, targeted therapy

## Abstract

**Purpose:** Locoregional therapies, such as transarterial embolization (TAE) and transarterial chemoembolization (TACE), are central to the treatment of unresectable hepatocellular carcinoma (HCC), particularly at the intermediate stage. However, there have been few large-scale real-world data comparisons of their effectiveness with that of systemic therapies. This study aimed to assess the relationships between different treatment modalities and all-cause mortality in a nationwide HCC cohort.

**Patients and methods:** We used the Taiwan National Health Insurance Research Database to recruit and identify 225,631 patients diagnosed with HCC between 2008 and 2021. The analyzed treatment modalities included hepatic resection, chemotherapy, targeted therapy, immunotherapy, TAE, and TACE. Cox proportional hazards models were applied to analyze the adjusted hazard ratios (HRs) for mortality.

**Results:** Both TAE (HR: 0.17) and TACE (HR: 0.17) were independently associated with significantly reduced mortality (*p* < 0.0001). In contrast, targeted therapy (HR: 6.17) and immunotherapy (HR: 5.84) were associated with increased mortality, probably because the selected patients had more advanced diseases. Older age and male sex were also independently associated with worse outcomes. There was no significant association between chemotherapy and mortality.

**Conclusion:** In this large, population-based, real-world cohort, TAE and TACE were significantly associated with better survival in patients with unresectable HCC, supporting their continued use as standard-of-care treatments in appropriately selected patients. The results highlight the need for multidisciplinary approaches to optimize advanced HCC outcomes.

## Introduction

As the fourth most common malignancy and the second leading cause of cancer-related mortality, hepatocellular carcinoma (HCC) remains a major public health burden in Taiwan.^1^ The Taiwan Cancer Registry reported 11,272 new HCC cases and 7,881 deaths in 2019, with a high mortality-to-incidence ratio reflecting the disease's aggressive nature and challenges in early detection.^2^ The proportion of cases attributable to non-viral etiologies, such as alcohol-related liver disease and metabolic dysfunction-associated steatotic liver disease, continues to rise despite nationwide initiatives, including universal hepatitis B vaccination, expanded access to antiviral therapy, and implementation of a national hepatitis C elimination program, that have significantly reduced the burden of virus-related HCC.^3^ This evolving epidemiological landscape reflects the sustained impact of HCC on the healthcare system. Particularly for patients with unresectable disease, curative options are limited, so continued efforts to improve treatment outcomes and reduce HCC-related mortality are needed.

Depending on the tumor stage, treatment approaches for HCC vary significantly, ranging from potentially curative interventions, such as hepatectomy, liver transplantation, and local ablative therapies, in early-stage disease to systemic therapies, including chemotherapy, targeted therapy, and immunotherapy in advanced stage disease.^4^,^5^ For patients with unresectable tumors, minimally invasive interventional radiology procedures, particularly transarterial embolization (TAE) and transarterial chemoembolization (TACE), have become essential therapeutic options.^6^ Specifically, in patients presenting with intermediate stage disease according to the Barcelona Clinic Liver Cancer (BCLC) staging system (stage B), characterized by multinodular tumors restricted to the liver without distant metastases or vascular invasion, TAE and TACE are the standard-of-care treatments.^6^ By selectively obstructing arterial blood flow, inducing ischemic necrosis and significant tumor shrinkage even without adjunctive chemotherapy, TAE alone effectively inhibits tumor growth.^7^ Integration of chemotherapy into embolization procedures (TACE) enhances therapeutic efficacy by providing targeted drug delivery and increased tumor cytotoxicity.^8^ These locoregional therapies substantially delay tumor progression, preserve residual liver function, and improve overall patient survival. Given their clinical significance, continual refinements of TAE and TACE are critical for optimizing patient outcomes and enhancing the quality of life for individuals with unresectable HCC.

Taiwan's National Health Insurance Research Database (NHIRD) is one of the largest population-based clinical databases in the world and a valuable source of real-world data. This robust database provides an opportunity to evaluate treatment outcomes across a wide spectrum of HCC cases, including both resectable and unresectable diseases. The study aim was to use data from the NHIRD to comprehensively assess the real-world effectiveness and safety of various therapeutic strategies, particularly TAE and TACE, as well as systemic therapies, such as chemotherapy, targeted therapy, and immunotherapy in patients with different stages of HCC. Another study goal was to provide large-scale, evidence-based insights to guide clinical decision-making and inform the development of updated treatment algorithms tailored to real-world practice in Taiwan. It is hoped that these findings will provide important evidence to inform and refine future treatment guidelines for HCC in Taiwan and other countries.

## Material and Methods

### Dataset Source

In this population-based study, we used data from the National Health Insurance Research Database of the Taiwan Ministry of Health and Welfare (NHIRD_MOHW), a comprehensive insurance claims database. The NHIRD_MOHW covers >99% of Taiwan's population and includes >95% of all healthcare-related data.^9^,^10^ This database provides extensive information on medical and pharmacy claims, diagnostic records, healthcare services, and prescription details, all derived from finalized payment claims submitted by healthcare providers.^11^,^12^ Disease diagnoses were cited according to the International Classification of Diseases, 9^th^ Revision, Clinical Modification (ICD-9-CM), and the 10^th^ Revision (ICD-10). For this study, we accessed the NHIRD_MOHW data from 2008 to 2021. Individual patients cannot be identified because the NHIRD_MOHW is an encrypted secondary dataset. All data were fully de-identified and could not be traced back to individual participants. Therefore, informed consent was not applicable, and a waiver of informed consent was obtained from the institutional review board prior to the commencement of the study. Approval was obtained from the Tri-Service General Hospital Joint Institutional Review Board (Approval number: TSGHIRB No. C202405196).

### Study sample and treatments

The study sample comprised patients diagnosed with HCC between 2008 and 2021. A cohort was identified by the ICD-9-CM codes 155.0, 155.1, and 155.2, the ICD-10-CM codes C22.0, C22.2, C22.7, and C22.8, and the records from the Registry for Catastrophic Illness in the NHIRD_MOHW between January 1, 2008, and December 31, 2021. The treatments analyzed in this study included hepatic operation (ICD-9-CM codes 50.2, 50.21, 50.22, 50.23, 50.24, 50.25, 50.26, 50.29, 50.3, 50.4), chemotherapy (using cisplatin, doxorubicin, mitomycin-C, or fluorouracil), targeted therapies (sorafenib or lenvatinib), immunotherapies (nivolumab, bevacizumab, pembrolizumab, or atezolizumab), TAE (ICD-9-CM codes 99.29, 88.47), and TACE (ICD-9-CM codes 99.25, 99.29, 88.47).

### Outcome measurements and covariates

The primary study outcome was all-cause mortality assessed by Kaplan-Meier survival analysis over 13-years of follow-up. The patient death dates were obtained from the national death registry in the NHIRD_MOHW. All eligible patients were followed up from the study entry date until death, withdrawal from the NHIRD, or the end of 2021, whichever came first. The tracked comorbidities included lipid abnormalities (ICD-9-CM: 272; ICD-10-CM: E7130, E752, E753, E755-E756, E77, E780-E789, E881, E882, and E8889); hypertension (ICD-9-CM: 401-405; ICD-10-CM: I10-I15), gout (ICD-9-CM: 274; ICD-10-CM: M10, M1A); ischemic heart diseases (ICD-9-CM: 410-414 and 557; ICD-10-CM: I20-I25); cerebrovascular disease (ICD-9-CM: 430-438; ICD-10-CM: I60-I69); congestive heart failure (ICD-9-CM: 398.91, 402.01, 402.11, 402.91, 404.01, 404.03, 404.11, 404.13, 404.91, 404.93, 425.4, 425.5, 425.7, 425.8, 425.9, and 428; ICD-10-CM: I0981, I110, I130, I132, I420, I422, I425, I428, I429, I426, I43, I427, I429, and I50); depression (ICD-9-CM: 3004, 2962, 2963, 3090,3091, 311; ICD-10-CM: F341, F32, F33, F4321, F329); and cancer (ICD-9-CM:140-165, 170-195, 200-208, 2386, 196-199; ICD-10-CM: Z51, C00, C02-C16, C7A, C18-C26, C30-C34, C37-C57, C60, C62-C86, C88, C96, D03, D3A, D47Z9, E3122, J91, R18).

### Statistical analysis

We performed the Chi-squared test to compare baseline characteristics, including sex and treatment types, between surviving and deceased patients with HCC. To evaluate the relationship between the different treatments and mortality, we performed multivariate Cox proportional hazards regression models, adjusting for all relevant covariates. The results are expressed as hazard ratios (HRs) with 95% confidence intervals (CIs). The multivariable models were adjusted for factors, such as age, sex, hypertension, dyslipidemia, gout, cerebrovascular disease, congestive heart failure, ischemic heart disease, depression, and cancer. SAS (version 9.4; SAS Institute, Cary, NC, USA) was used to perform all statistical analyses. Statistical significance was accepted for values of *p* < 0.05.

## Results

Table 1 presents the baseline characteristics and treatment distributions of 225,631 HCC patients. The cohort had a mean age of 64.17 years, and the majority of patients were male (66.92%). Nearly all patients received chemotherapy (99.8%), and almost all underwent targeted therapy (98.64%) and immunotherapy (98.58%). The cohort's overall mortality rate was 70.97%.

Table 2 compares the baseline characteristics between patients who survived and those who did not. Significant differences were observed across all variables, including sex and treatment modalities. Male patients, as well as those receiving targeted therapy, immunotherapy, or hepatectomy, were more prevalent in the death group (*p* < 0.0001). Conversely, patients who underwent TAE or TACE were more frequently found in the non-death group, suggesting that these procedures may be protective. These findings suggest that both sex and treatment type have critical roles in determining HCC survival outcomes.

Table 3 presents the crude and adjusted hazard ratios (HRs) for death among HCC patients. After adjustment for confounding factors, increasing age was significantly associated with higher mortality risk (HR: 1.031, 95% CI: 1.031-1.032, *p* < 0.0001). The risk of mortality was lower for female patients than for males (HR: 0.869, 95% CI: 0.86-0.878, *p* < 0.0001). There was no significant association between chemotherapy and mortality (HR: 0.841, 95% CI: 0.664-1.065, *p* = 0.1512). In contrast, targeted therapy (HR: 6.168, *p* < 0.0001) and immunotherapy (HR: 5.841, 95% CI: 5.381-6.34, *p* < 0.0001) were linked to significantly higher mortality. Procedures, such as TAE, TACE, and hepatectomy, were associated with significantly lower mortality risk (all *p* < 0.0001). These protective effects persisted across different combinations of treatments, such as TAE or TACE with chemotherapy, targeted therapy, or immunotherapy, indicating that these interventions significantly attenuated the risk of death in the patients with HCC (Table 4).

## Discussion

Utilizing NHIRD-population based cohort study, we identified several important findings regarding treatment outcomes among the patients with HCC. First, both TAE and TACE were associated with a significant protective effect, demonstrating their ability to reduce HCC-related mortality in real-world settings. This finding reinforces the essential role of these locoregional therapies for improving survival among patients with unresectable, intermediate stage (BCLC stage B) disease. Second, male sex and older age were independently associated with a higher risk of death, emphasizing the prognostic influence of demographic factors. Third, among the systemic treatment options, chemotherapy showed no significant association with mortality, whereas both targeted therapy and immunotherapy were linked to significantly higher mortality. This finding probably reflects the predominant use of targeted therapy and immunotherapy in patients with more severe disease, highlighting the complexities of treatment selection in late-stage HCC.

The therapeutic value of TAE and TACE is their ability to achieve effective intrahepatic tumor control through precise, image-guided vascular intervention, making them especially important for managing unresectable HCC. These procedures selectively occlude tumor-feeding arteries under fluoroscopic guidance, enabling interventional radiologists to directly deliver treatment to the tumor while preserving the surrounding healthy tissue.^13^ TAE uses mechanical embolization to induce ischemia and necrosis by cutting off arterial blood flow, whereas TACE combines this with localized chemotherapy to enhance cytotoxic effects and tumor regression.^14^ Durable local control is achieved via this dual approach. The role of interventional radiology in these procedures highlights the need for coordinated multidisciplinary care in unresectable HCC. Consistent with these mechanisms, our real-world findings showed that both TAE and TACE significantly reduced mortality in patients presenting with unresectable HCC, reinforcing their value for improving survival outcomes in clinical practice.

Several previous studies have identified the clinical benefit of transarterial therapies in patients presenting with unresectable HCC, particularly at the intermediate stage. In a landmark randomized controlled trial, Llovet et al. reported that TACE significantly improved the overall survival in patients with BCLC stage B HCC, establishing it as a standard-of-care treatment in this population^15^. Similarly, Lo et al. demonstrated that TAE alone compared with supportive care was also associated with survival benefits, highlighting its value as an effective locoregional therapy.^16^ These findings, corroborated by subsequent meta-analyses, are the basis for the current guideline recommendations. However, particularly in patients with a large tumor burden or compromised liver function, the application of TAE and TACE has a risk of hepatic decompensation. Therefore, these treatments are typically recommended for individuals with results showing well-preserved liver function and tumors restricted to the liver, without evidence of extrahepatic spread or vascular invasion. Accordingly, current clinical guidelines endorse the use of TAE and TACE primarily in patients categorized as BCLC stage B since the therapeutic benefit outweighs the potential for treatment-related adverse effects in these patients.^17^,^18^

Age and sex have been consistently identified as important prognostic determinants in HCC. Many studies have shown that increasing age is associated with poorer survival outcomes. Kanneganti et al. demonstrated that mortality was significantly higher in patients aged ≥65 years old than in younger individuals, even among those with early-stage disease who underwent curative treatments.^19^ Similarly, a large population-based study using SEER-Medicare data by White et al. reported that older age independently predicted decreased overall survival in patients with HCC after adjustment for treatment modality and comorbidity burden.^20^ Sex-related disparities have also been well documented. Men are disproportionately affected by HCC, with incidence rates 4-to 8-fold higher than those observed in women.^21^ Analysis of SEER data also revealed that male sex was independently associated with increased risks of postoperative hepatic decompensation, major complications, and mortality.^22^ These findings highlight the prognostic relevance of demographic factors and the necessity of incorporating age and sex into individualized treatment decisions. Consistent with previous literature, our real-world cohort analysis also found that advanced age and male sex were independently associated with increased mortality, further supporting the importance of demographic stratification in the clinical risk assessment and management of HCC.

In recent years, systemic treatment options for unresectable HCC have evolved considerably, particularly with the availability of immune checkpoint inhibitors and targeted therapies.^23^-^27^ Immunotherapeutic agents, such as nivolumab, pembrolizumab, and the combination of atezolizumab and bevacizumab, have shown promising efficacy by reinvigorating anti-tumor immune responses and improving overall survival in patients with advanced stage disease.^23^-^25^ Concurrently, targeted therapies, including multikinase inhibitors (such as sorafenib, lenvatinib, and regorafenib), disrupt tumor angiogenesis and cellular proliferation, so they remain important in both first-line and subsequent treatment settings for patients ineligible for locoregional interventions.^26^,^27^ These agents are typically reserved for patients with advanced or progressive unresectable HCC and have demonstrated clinical benefit in some randomized controlled trials. However, their effectiveness in real-world settings may be influenced by baseline liver function, tumor burden, and patient comorbidities, which differ from the controlled environments of clinical trials. In our real-world cohort, systemic chemotherapy was not significantly associated with mortality, whereas both targeted therapy and immunotherapy were associated with higher mortality. This association probably reflects their preferential use in patients with more aggressive disease biology or advanced stage tumors, underscoring the challenges of treatment selection and outcome interpretation in routine clinical practice.

Our findings are consistent with current guideline recommendations. The Taiwan Liver Cancer Association (TLCA, 2023 update) and the 2025 European Society for Medical Oncology (ESMO) guidelines recommend TAE and TACE as first-line therapies for unresectable, intermediate-stage HCC (BCLC stage B) because of their ability to improve survival while preserving liver function. 28,29. The significantly lower hazard ratios observed in our nationwide cohort provide strong real-world evidence reinforcing these recommendations at a population level. In contrast, targeted therapy and immunotherapy are primarily indicated for advanced-stage disease according to these same guidelines, which explain their association with higher mortality in our study and should not be interpreted as reduced therapeutic efficacy.^28^,^29^. These results support guideline-directed treatment stratification and underscore the importance of stage-specific management.

Our study had several notable strengths. First, it is among the largest population-based cohorts of HCC worldwide, including a total of 225,631 patients diagnosed with HCC over 13 years. This large sample enhances the generalizability and statistical power of our findings. Second, we comprehensively evaluated a broad spectrum of real-world treatment strategies for unresectable HCC, including chemotherapy, immunotherapy, targeted therapy, and TAE and TACE alone or in their various combinations, which enabled a robust assessment of their individual and combined effects on patient outcomes. This comprehensive approach reflects the complex treatment landscape of unresectable HCC and contributes meaningful insights into clinical practice.

Despite these study strengths, several limitations should be considered. First, the retrospective observational design inherently precludes the establishment of causality and is susceptible to unmeasured confounding. Second, treatment selection bias may have influenced the observed associations because advanced or more localized disease was probably less common in the patients receiving TAE or TACE than in those receiving systemic therapies, such as targeted agents or immune checkpoint inhibitors, which are typically reserved for patients with more advanced, aggressive, or refractory disease. Consequently, differences in baseline tumor burden and disease biology could have affected survival outcomes regardless of the treatment modality. Third, although the NHIRD provides comprehensive population-level data, it lacks detailed clinical parameters, such as tumor staging, histopathological characteristics, performance status (e.g., ECOG), and liver functional reserve indicators, including Child-Pugh class. These omissions can introduce residual confounding, which limits our ability to perform nuanced risk adjustment. Fourth, although TAE and TACE are commonly used for unresectable or intermediate-stage HCC, specific information required to determine surgical resectability was not available in our dataset. Therefore, we could not differentiate resectable from unresectable cases with certainty. Fifth, the identification of TAE and TACE procedures was based on ICD-9/10 and procedure codes, which are commonly used and validated in previous NHIRD-based studies. However, the possibility of coding errors or misclassification cannot be entirely excluded. Sixth, the notably low adjusted hazard ratios observed for TAE and TACE may partly result from confounding by indication. Patients who are selected for these locoregional procedures generally have better liver function or overall health status than those receiving systemic therapies, which may exaggerate the apparent protective effects despite statistical adjustment. Seventh, the high prevalence of chemotherapy, targeted therapy, and immunotherapy in our cohort may partly result from overlapping claims or misclassification in the coding of therapeutic categories, which could have led to overestimation of treatment frequencies and should be interpreted with caution when comparing hazard ratios across modalities.

## Conclusion

This large population-based study provides robust real-world evidence supporting the protective effect of TAE and TACE in reducing mortality among patients with unresectable HCC. Despite the advent of novel systemic therapies, our findings reaffirm the central role of these locoregional treatments within multidisciplinary care. Given their survival benefits and liver-preserving potential, TAE and TACE should remain key therapeutic options for appropriately selected patients. Further prospective studies incorporating detailed clinical and biomarker data are needed to refine patient selection and optimize individualized treatment strategies.

## Figures and Tables

**Table 1:** Demographics of the Patients with Hepatocellular Carcinoma (n = 225,631)

Variable	n/mean	%/±SD
Age	64.17	13.21
Sex		
Male	150,851	66.92
Female	74,574	33.08
Chemotherapy		
No	444	0.2
Yes	225,187	99.8
Targeted therapy		
No	3079	1.36
Yes	222,552	98.64
Immunotherapy		
No	3198	1.42
Yes	222,433	98.58
TAE		
No	212,593	94.22
Yes	13,038	5.78
TACE		
No	212,556	94.21
Yes	13,075	5.79
Hepatectomy		
No	172,638	76.51
Yes	52,993	23.49
Death		
No	65,497	29.03
Yes	160,134	70.97
TAE + Chemotherapy		
No	212,593	94.22
Yes	13,038	5.78
TACE + Chemotherapy		
No	212,556	94.21
Yes	13,075	5.79
Hepatectomy + Chemotherapy		
No	172,638	76.51
Yes	52,993	23.49
TAE+ Targeted therapy		
No	212,593	94.22
Yes	13,038	5.78
TACE+ Targeted therapy		
No	212,559	94.21
Yes	13,072	5.79
Hepatectomy + Targeted therapy		
No	172,638	76.51
Yes	52,993	23.49
TAE+ Immunotherapy		
No	212,593	94.22
Yes	13,038	5.78
TACE+ Immunotherapy		
No	212,559	94.21
Yes	13,072	5.79
Hepatectomy + Immunotherapy		
No	172,638	76.51
Yes	52,993	23.49
TAE + Chemotherapy+ Targeted therapy		
No	212,593	94.22
Yes	13,038	5.78
TACE + Chemotherapy+ Targeted therapy		
No	212,559	94.21
Yes	13,072	5.79
Hepatectomy + Chemotherapy+ Targeted therapy		
No	172,638	76.51
Yes	52,993	23.49
TAE + Chemotherapy + Immunotherapy		
No	212,593	94.22
Yes	13,038	5.78
TACE + Chemotherapy + Immunotherapy		
No	212,559	94.21
Yes	13,072	5.79
Hepatectomy + Chemotherapy + Immunotherapy		
No	172,638	76.51
Yes	52,993	23.49
TAE+ Targeted therapy + Immunotherapy		
No	212,593	94.22
Yes	13,038	5.78
TACE+ Targeted therapy + Immunotherapy		
No	212,559	94.21
Yes	13,072	5.79
Hepatectomy + Targeted therapy + Immunotherapy		
No	172,638	76.51
Yes	52,993	23.49
TAE + Chemotherapy+ Targeted therapy + Immunotherapy		
No	212,593	94.22
Yes	13,038	5.78
TACE + Chemotherapy + Targeted therapy + Immunotherapy		
No	212,559	94.21
Yes	13,072	5.79
Hepatectomy + Chemotherapy+ Targeted therapy + Immunotherapy		
No	172,638	76.51
Yes	52,993	23.49

**Table 2:** Patients with Hepatocellular Carcinoma: Non-death and Death (n = 225,631)

Variable	Non-death (n = 65,497)		Death (n = 160,134)		*p* Value
	n/means	%/±SD	n/means	%/±SD	
Sex					<0.0001
Male	42,745	65.31	108,106	67.58	
Female	22,702	34.69	51,872	32.42	
Chemotherapy					0.0005
No	95	0.15	349	0.22	
Yes	65,402	99.85	159,785	99.78	
Targeted therapy					<0.0001
No	2,202	3.36	877	0.55	
Yes	63,295	96.64	159,257	99.45	
Immunotherapy					<0.0001
No	2247	3.43	951	0.59	
Yes	63,250	96.57	159,183	99.41	
TAE					<0.0001
No	56,803	86.73	155,790	97.29	
Yes	8694	13.27	4344	2.71	
TACE					<0.0001
No	56,804	86.73	155,752	97.26	
Yes	8,693	13.27	4,382	2.74	
Hepatectomy					<0.0001
No	37,415	57.12	135,223	84.44	
Yes	28,082	42.88	24,911	15.56	
TAE+Chemotherapy					<0.0001
No	56,803	86.73	155,790	97.29	
Yes	8,694	13.27	4,344	2.71	
TACE+Chemotherapy					<0.0001
No	56,804	86.73	155,752	97.26	
Yes	8,693	13.27	4,382	2.74	
Hepatectomy+Chemotherapy					<0.0001
No	37,415	57.12	135,223	84.44	
Yes	28,082	42.88	24,911	15.56	
TAE+ Targeted therapy					<0.0001
No	56,803	86.73	155,790	97.29	
Yes	8,694	13.27	4,344	2.71	
TACE+ Targeted therapy					<0.0001
No	56,807	86.73	155,752	97.26	
Yes	8,690	13.27	4,382	2.74	
Hepatectomy + Targeted therapy					<0.0001
No	37,415	57.12	135,223	84.44	
Yes	28,082	42.88	24,911	15.56	
TAE+ Immunotherapy					<0.0001
No	56,803	86.73	155,790	97.29	
Yes	8,694	13.27	4,344	2.71	
TACE+ Immunotherapy					<0.0001
No	56,807	86.73	155,752	97.26	
Yes	8,690	13.27	4,382	2.74	
Hepatectomy + Immunotherapy					<0.0001
No	37,415	57.12	135,223	84.44	
Yes	28,082	42.88	24,911	15.56	
TAE + Chemotherapy + Targeted therapy					<0.0001
No	56,803	86.73	155,790	97.29	
Yes	8,694	13.27	4,344	2.71	
TACE + Chemotherapy + Targeted therapy					<0.0001
No	56,807	86.73	155,752	97.26	
Yes	8,690	13.27	4,382	2.74	
Hepatectomy + Chemotherapy+ Targeted therapy					<0.0001
No	37,415	57.12	135,223	84.44	
Yes	28,082	42.88	24,911	15.56	
TAE + Chemotherapy + Immunotherapy					<0.0001
No	56,803	86.73	155,790	97.29	
Yes	8,694	13.27	4,344	2.71	
TACE + Chemotherapy + Immunotherapy					<0.0001
No	56,807	86.73	155,752	97.26	
Yes	8,690	13.27	4,382	2.74	
Hepatectomy + Chemotherapy + Immunotherapy					<0.0001
No	37,415	57.12	135,223	84.44	
Yes	28,082	42.88	24,911	15.56	
TAE+ Targeted therapy + Immunotherapy					<0.0001
No	56,803	86.73	155,790	97.29	
Yes	8,694	13.27	4,344	2.71	
TACE+ Targeted therapy + Immunotherapy					<0.0001
No	56,807	86.73	155,752	97.26	
Yes	8,690	13.27	4,382	2.74	
Hepatectomy + Targeted therapy + Immunotherapy					<0.0001
No	37,415	57.12	135,223	84.44	
Yes	28,082	42.88	24,911	15.56	
TAE + Chemotherapy+ Targeted therapy + Immunotherapy					<0.0001
No	56,803	86.73	155,790	97.29	
Yes	8,694	13.27	4,344	2.71	
TACE + Chemotherapy + Targeted therapy + Immunotherapy					<0.0001
No	56,807	86.73	155,752	97.26	
Yes	8,690	13.27	4,382	2.74	
Hepatectomy + Chemotherapy + Targeted therapy + Immunotherapy					<0.0001
No	37,415	57.12	135,223	84.44	
Yes	28,082	42.88	24,911	15.56	

**Table 3:** Crude and Adjusted Hazard Ratios of Death among Patients with Hepatocellular Carcinoma across Major Treatment Modalities

	Crude HR(95% CI)			*p* Value	Adjusted HR (95% CI)			*p* Value
Age	1.024	1.024	1.025	<0.0001	1.031	1.031	1.032	<0.0001
Sex								
Male		ref				ref		
Female	0.937	0.927	0.947	<0.0001	0.869	0.86	0.878	<0.0001
Chemotherapy								
No		ref				ref		
Yes	0.660	0.525	0.829	0.0004	0.841	0.664	1.065	0.1512
Targeted therapy								
No		ref				ref		
Yes	6.325	5.845	6.844	<0.0001	6.168	5.669	6.712	<0.0001
Immunotherapy								
No		ref				ref		
Yes	5.952	5.514	6.425	<0.0001	5.841	5.381	6.34	<0.0001
TAE								
No		ref				ref		
Yes	0.182	0.175	0.189	<0.0001	0.171	0.164	0.178	<0.0001
TACE								
No		ref				ref		
Yes	0.184	0.177	0.191	<0.0001	0.172	0.166	0.179	<0.0001
Hepatectomy								
No		ref				ref		
Yes	0.245	0.240	0.251	<0.0001	0.262	0.257	0.268	<0.0001

Adjusted for covariate factors, including age, sex, hypertension, lipid abnormalities, gout, ischemic heart disease, cerebrovascular disease, congestive heart failure, depression, and cancer.

**Table 4:** Crude and Adjusted Hazard Ratios of Death in Patients with Hepatocellular Carcinoma across Different Combinations of Treatments

	Crude HR (95% CI)			*p* Value	Adjusted HR (95% CI)			*p* Value
TAE + Chemotherapy								
No		ref				ref		
Yes	0.182	0.175	0.189	<0.0001	0.171	0.164	0.178	<0.0001
TACE + Chemotherapy								
No		ref				ref		
Yes	0.184	0.177	0.191	<0.0001	0.172	0.166	0.179	<0.0001
Hepatectomy + Chemotherapy								
No		ref				ref		
Yes	0.245	0.24	0.251	<0.0001	0.262	0.257	0.268	<0.0001
TAE + Targeted therapy								
No		ref				ref		
Yes	0.182	0.175	0.189	<0.0001	0.171	0.164	0.178	<0.0001
TACE+ Targeted therapy								
No		ref				ref		
Yes	0.184	0.177	0.191	<0.0001	0.173	0.166	0.179	<0.0001
Hepatectomy + Targeted therapy								
No		ref				ref		
Yes	0.245	0.24	0.251	<0.0001	0.262	0.257	0.268	<0.0001
TAE + Immunotherapy								
No		ref				ref		
Yes	0.182	0.175	0.189	<0.0001	0.171	0.164	0.178	<0.0001
TACE + Immunotherapy								
No		ref				ref		
Yes	0.184	0.177	0.191	<0.0001	0.173	0.166	0.179	<0.0001
Hepatectomy + Immunotherapy								
No		ref				ref		
Yes	0.245	0.24	0.251	<0.0001	0.262	0.257	0.268	<0.0001
TAE + Chemotherapy + Targeted therapy								
No		ref				ref		
Yes	0.182	0.175	0.189	<0.0001	0.171	0.164	0.178	<0.0001
TACE + Chemotherapy + Targeted therapy								
No		ref				ref		
Yes	0.184	0.177	0.191	<0.0001	0.173	0.166	0.179	<0.0001
Hepatectomy + Chemotherapy + Targeted therapy								
No		ref				ref		
Yes	0.245	0.24	0.251	<0.0001	0.262	0.257	0.268	<0.0001
TAE + Chemotherapy + Immunotherapy								
No		ref				ref		
Yes	0.182	0.175	0.189	<0.0001	0.171	0.164	0.178	<0.0001
TACE + Chemotherapy + Immunotherapy								
No		ref				ref		
Yes	0.184	0.177	0.191	<0.0001	0.173	0.166	0.179	<0.0001
Hepatectomy + Chemotherapy + Immunotherapy								
No		ref				ref		
Yes	0.245	0.24	0.251	<0.0001	0.262	0.257	0.268	<0.0001
TAE + Targeted therapy + Immunotherapy								
No		ref				ref		
Yes	0.182	0.175	0.189	<0.0001	0.171	0.164	0.178	<0.0001
TACE + Targeted therapy + Immunotherapy								
No		ref				ref		
Yes	0.184	0.177	0.191	<0.0001	0.173	0.166	0.179	<0.0001
Hepatectomy + Targeted therapy + Immunotherapy								
No		ref				ref		
Yes	0.245	0.240	0.251	<0.0001	0.262	0.257	0.268	<0.0001
TAE + Chemotherapy + Targeted therapy + Immunotherapy								
No		ref				ref		
Yes	0.182	0.175	0.189	<0.0001	0.171	0.164	0.178	<0.0001
TACE + Chemotherapy + Targeted therapy + Immunotherapy								
No		ref				ref		
Yes	0.184	0.177	0.191	<0.0001	0.173	0.166	0.179	<0.0001
Hepatectomy + Chemotherapy + Targeted therapy + Immunotherapy								
No		ref				ref		
Yes	0.245	0.240	0.251	<0.0001	0.262	0.257	0.268	<0.0001

Adjusted for covariate factors, including age, sex, hypertension, lipid abnormalities, gout, ischemic heart disease, cerebrovascular disease, congestive heart failure, depression, and cancer.

## Abbreviations

BCLC: Barcelona Clinic Liver Cancer
Cis: confidence intervals
HCC: Hepatocellular carcinoma
HRs: hazard ratios
ICD-9-CM: International Classification of Diseases, 9^th^ Revision, Clinical Modification
ICD-10: International Classification of Diseases, 10^th^ Revision
NHIRD_MOHW: National Health Insurance Research Database of the Taiwan Ministry of Health and Welfare
NHIRD: National Health Insurance Research Database
TAE: transarterial embolization
TACE: Transarterial chemoembolization
